# Impact on the microstructure of deep gray matter in unvaccinated patients after moderate-to-severe COVID-19: insights from MRI T1 mapping

**DOI:** 10.1186/s41747-025-00598-7

**Published:** 2025-07-05

**Authors:** Masia Fahim, Elke Hattingen, Alina Jurcoane, Jan R. Schüre, Svenja Klinsing, Julia Koepsell, Kolja Jahnke, Michael W. Ronellenfitsch, Ulrich Pilatus, Maria J. G. T. Vehreschild, Ralf Deichmann, Christophe T. Arendt

**Affiliations:** 1 Institute of Neuroradiology, University Hospital, Goethe University Frankfurt, Frankfurt am Main, Germany; 2 Department of Neurology, University Hospital, Goethe University Frankfurt, Frankfurt am Main, Germany; 3 Department of Internal Medicine, Infectious Diseases, University Hospital, Goethe University Frankfurt, Frankfurt am Main, Germany; 4 Dr. Senckenberg Institute of Neurooncology, University Hospital, Goethe University Frankfurt, Frankfurt am Main, Germany; 5 Brain Imaging Center, Goethe University Frankfurt, Frankfurt am Main, Germany

**Keywords:** Brain mapping, COVID-19, Gray matter, Magnetic resonance imaging, SARS-CoV-2

## Abstract

**Background:**

To determine changes in quantitative T1 relaxation times (qT1) in deep gray matter in patients recovered from coronavirus disease 2019 (COVID-19).

**Methods:**

Unvaccinated COVID-19 participants ≥ 3 months after seropositivity and age- and sex-matched controls were examined using 3-T magnetic resonance imaging. Bilateral measures of thalamus, pallidum, putamen, caudate and accumbens nuclei, and hippocampus were extracted from qT1 maps after automated segmentation. Baseline characteristics and results of tests assessing neurological functions (standardized exam), ability to smell (4-Item Pocket Smell Test), depression (Beck Depression Inventory-II), sleepiness (Epworth Sleepiness Scale), sleep quality (Pittsburgh Sleep Quality Index), health-related quality of life (EQ-5D), and cognitive performance (Montreal Cognitive Assessment) were evaluated.

**Results:**

One hundred forty-five subjects (median age, 46 years; 73 females) were included (11/2020–12/2021): 69 recovered after COVID-19 and 76 controls (age, *p* = 0.532; sex, *p* = 0.799), without significant differences in qT1 values overall (all *p*-values > 0.050). Subgroup analysis of participants aged ≥ 40 (age, *p* = 0.675; sex, *p* = 0.447) revealed higher qT1 values in previously hospitalized COVID-19 subjects (23/69) compared to controls (47/76) in left and right caudate nuclei (*p* = 0.009; *p* = 0.027), left accumbens nucleus (*p* = 0.017), right putamen (*p* = 0.041), and right hippocampus (*p* = 0.020). No correlations were found with macroscopic imaging findings, pre-existing conditions, time since COVID-19 diagnosis, inpatient treatment duration, or test results.

**Conclusion:**

T1 mapping revealed microstructural changes in striatal and hippocampal regions of unvaccinated individuals aged ≥ 40 who recovered from moderate-to-severe COVID-19 during the pre-Omicron era.

**Relevance statement:**

This study elucidates brain involvement following severe acute respiratory syndrome coronavirus 2 (SARS-CoV-2) infection, underscoring the need for further longitudinal analyses to assess the potential reversibility, stability or deterioration of these findings.

**Key Points:**

- We hypothesized altered T1 relaxation times in deep gray matter after COVID-19.
- Unvaccinated participants ≥ 40 years exhibited higher striatal, hippocampal qT1 after moderate-to-severe COVID-19.
- No qT1 correlations were found with hospitalization duration, pre-existing conditions, or neuro-(psycho)logical tests.

**Graphical Abstract:**

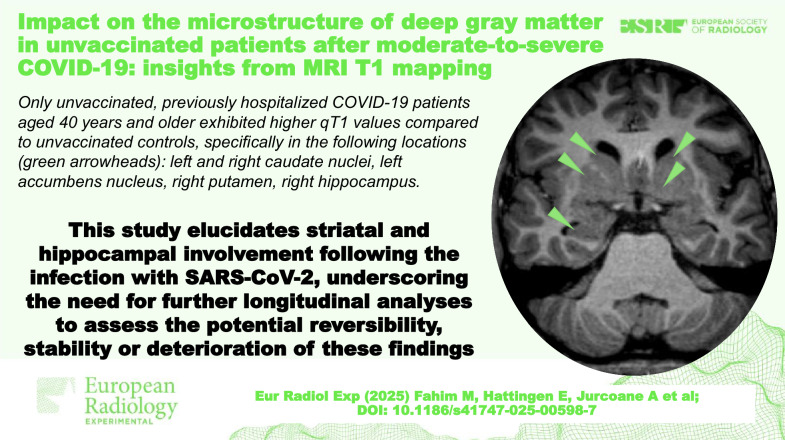

## Background

Acute and prolonged neurological symptoms are among the main sequelae of coronavirus disease 2019 (COVID-19), caused by the severe acute respiratory syndrome coronavirus 2 (SARS-CoV-2) infection [1]. During the acute phase, the central nervous system (CNS) can be indirectly affected by vascular complications, particularly embolic cerebral infarction and intracerebral hemorrhage, as well as by the systemic inflammatory response [2]. However, the mechanisms and extent of direct CNS infection with SARS-CoV-2 remain controversial. Olfactory dysfunction, the most common initial symptom, arises from direct infection of the olfactory epithelium [3]. Apart from the dense expression of the angiotensin-converting enzyme 2 in this region, its concentration within the CNS itself appears to be low and dispersed. Evidence of direct viral involvement is limited to the detection of low levels of viral ribonucleic acid and rare viral antigens in cranial nerves and scattered brainstem cells [4]. The olfactory bulb and nerve have therefore been discussed as a gateway for the virus to enter the brain [3]. Such transneural circulation has been demonstrated in murine models of coronavirus infection and is a well-known mechanism for human neurotropic viruses [5]. Another potential mechanism of CNS involvement is through parainfectious acute disseminated inflammation, which can occur with any viral infection [6]. It remains unclear whether, and to what extent, the coronavirus induces sustained inflammation in the CNS [4, 7]. Indeed, infections with various types of respiratory viruses have been associated with CNS disorders, as both viral proteins and corresponding signal abnormalities on magnetic resonance imaging (MRI) were predominantly observed in deep gray matter (GM) structures such as the hippocampus, basal ganglia, and thalamus [8–10].

In addition to serum and cerebrospinal fluid analyses, MRI is the method of choice for depicting neuroinflammation *in vivo*. While conventional MRI is insensitive to subtle inflammatory activity, such as that seen in multiple sclerosis, T1 and T2 relaxation times were significantly altered in normal-appearing brain tissue of patients with multiple sclerosis [11, 12]. This standardized procedure enables inferences about the microarchitecture of the region of interest to be drawn from quantitative MR relaxometry and serves as a sensitive method to detect microstructural changes in various neurological diseases [13]. Since quantitative mapping of T1 relaxation times (qT1) provides information solely on tissue parameters that reflect T1 relaxation times, it is not biased by other physical properties or the individual scanner hardware. Prolongation of qT1 can result from replacement of normal tissue, such as reactive gliosis, axonal or neuronal loss, or edema/inflammation [14–16]. In individuals with neurological long-COVID symptoms, conventional MRI is typically unremarkable or shows only nonspecific changes [17, 18]. To our knowledge, no study to date has comprehensively evaluated qT1 in the brains of patients who have recovered from the SARS-CoV-2 infection.

We hypothesized that qT1 in deep GM structures would differ significantly between recovered COVID-19 patients and age- and sex-matched, SARS-CoV-2-antibody-negative controls, with both groups unvaccinated.

## Methods

### Standard protocol approvals, registrations and patient consents

The study was approved by the Ethics Committee of the Faculty of Medicine at Goethe University Frankfurt, Germany (20-838) and registered at the German Clinical Trials Register (DRKS00023880). In this prospective, observational, exploratory study, patients aged ≥ 18 years who had tested positive for SARS-CoV-2 by reverse transcription-polymerase chain reaction test on naso-/oropharyngeal specimens at least 3 months earlier were consecutively included via the local Department of Infectious Diseases. The patient groups were prespecified based on whether they endured the disease at home (group 2) or in the hospital (group 3). The control subjects (group 1) were age- and sex-matched adult volunteers who had not experienced COVID-19-related symptoms. As asymptomatic infections with SARS-CoV-2 might have occurred in this group of unvaccinated controls, additional serological testing of antiviral immunoglobulin G and M (SARS-CoV-2 rapid Antibody Test, Roche Diagnostics, Mannheim, Germany) was performed immediately prior to MRI scanning in each subject to rule out previous COVID-19 [19]. Exclusion criteria were prior vaccination against SARS-CoV-2, known previous or prevailing neuroinflammatory or systemic inflammatory diseases, the permanent use of immunosuppressive drugs, pregnancy, and contraindications to MRI. Each participant signed an informed consent form for research. The study was conducted in accordance with the ethical guidelines for research involving human subjects, as outlined in the Declaration of Helsinki.

### Clinical and neurological assessment

Prior to the acquisition of MRI datasets, all study participants completed a questionnaire about pre-existing conditions and COVID-19-related symptoms. The latter were further categorized into symptoms present during infection and/or persistent at the time of MRI. All subjects received a standardized exam from experienced neurologists. Smell testing was performed with eight different odors using the 4-Item Pocket Smell Test, versions A and B (Sensonics Inc., Haddon Heights, NJ, USA) [20]. Depression, sleepiness, quality of sleep, and health-related quality of life were assessed using the Beck Depression Inventory-II [21], Epworth Sleepiness Scale [22], Pittsburgh Sleep Quality Index [23] and EQ-5D [24]. For cognitive screening, the Montreal Cognitive Assessment (version 8.1) [25] was conducted.

### Magnetic resonance imaging protocol and image analysis

MRI examinations were acquired on a whole-body 3-T scanner (MAGNETOM Prisma, Siemens Healthineers, Erlangen, Germany) with a body coil for radiofrequency transmission and a 20-channel phased-array head coil for radiofrequency reception. The sequence parameters for qT1 mapping are shown in the Supplementary Table S1, and further steps of qT1 mapping are detailed in the Supplementary Material. In addition, an axial two-dimensional fluid-attenuated inversion recovery (FLAIR) sequence was acquired with fat saturation, 3-mm slice thickness, field of view of 210 × 210 × 149 mm³, repetition time / echo time / inversion time of 9,000 / 81 / 2,500 ms, flip angle (α) of 150°, and acquisition time of 4:14 min:s.

Computation of qT1 maps and data processing were performed with custom-made programs using MATLAB (MathWorks) and FSL (FMRIB, Software Library version 5.0, https://fsl.fmrib.ox.ac.uk/fsl) [26]. Automatic segmentation of the anatomical structures, including deep GM, was performed on synthetic magnetization-prepared rapid acquisition gradient-echo images of each subject (native space) using FSL with its tool FIRST. The following virtual acquisition parameters were utilized: 1-mm isotropic resolution, field of view of 256 × 224 × 160 mm^3^, repetition time / echo spacing / inversion time of 1,900 / 8.1 / 900 ms, and α = 9°. Based on this segmentation tool, the relevant deep GM structures (*i.e*., left- and right-sided caudate nuclei, putamen, accumbens nuclei, pallidum, thalami, and hippocampi) underwent the exploratory analysis to test for differences in qT1 and volume values between groups. To minimize contamination of normal-appearing GM by macroscopic pathological T1 hypointensities (*e.g*., neighboring white matter hyperintensities (WMH), lacunes) and cerebrospinal fluid, we applied a threshold of 80% for extracting the GM structures from the partial volume estimates resulting from segmentation. Total intracranial volume (TIV) was calculated by summing the number of voxels of GM, white matter, and cerebrospinal fluid from the native space images, and then multiplying the total by the native voxel volume. Regional brain volumes were normalized by dividing each by the respective subject’s TIV, aiding in controlling for inter-individual variability in head size. ITK‐SNAP (version 3.6.0) was used for visualization, quality control of segmentation, and extraction of qT1 and volume values for each structure.

The interpretation of macrostructural brain abnormalities was performed visually on the synthetic magnetization-prepared rapid acquisition gradient-echo and FLAIR images by one experienced neuroradiologist (C.T.A.), blinded to the underlying clinical information. Chronic cerebral infarctions were defined as parenchymal defects isointense to cerebrospinal fluid and were further specified by localization, number, size, and origin, either from large vessel disease (territorial pattern) or small vessel disease (SVD) (nonterritorial/lacunar pattern). WMH were defined as hyperintense areas (> 2 mm) on FLAIR images and were classified based on the distribution pattern characteristic of SVD, defined by the presence of bilateral, mostly symmetrical lesions in the deep white matter [27, 28]. Deep WMH were graded using the Fazekas scale and by evaluating their number [29]. The scores and scaling criteria for deep WMH are described in Supplementary Table S2.

### Statistical analysis

Computations were performed using SPSS software (version 27.0, IBM SPSS Statistics, IBM Corporation). All tests were two-tailed, and *p*-values < 0.050 were considered statistically significant. The Kolmogorov-Smirnov test with Lilliefors correction was used to test continuous data for normality of distribution. Nonnormally distributed variables are presented as medians with interquartile ranges (IQR) in squared brackets [first quartile–third quartile]. Kruskal–Wallis, Wilcoxon–Mann–Whitney *U*, and Pearson’s chi-squared tests were used to assess differences between groups. To analyze differences in qT1 values between groups using the Wilcoxon–Mann–Whitney *U* test, each group of subjects (groups 1, 2, and 3) was further subdivided according to an age cutoff of 40 years (*stratum* 1, < 40 years old; *stratum* 2, ≥ 40 years old) to discriminate potential age-dependent microstructural alterations from SARS-CoV-2-associated changes. *Post hoc* correction for the computed *p*-values was performed using the Benjamini-Hochberg method, controlling for a 10% false discovery rate in multiple comparisons [30]. This was done separately for each hemisphere to account for anatomical left-right asymmetry and functional lateralization of the human brain (*i.e*., *n* = 12 structures) [31]. Odds ratios and their corresponding 95% confidence intervals were calculated using logistic regression models to determine associations between variables. Further details on the statistical analysis are provided in Supplementary Material.

## Results

Datasets of 145 unvaccinated subjects (median [IQR] age, 46 [33.5–53] years; 73 women) were included between November 2020 and December 2021. Of these, 69 participants had previously managed COVID-19 either at home (group 2) or in the hospital (group 3), and 76 served as age- and sex-matched controls (group 1). Demographics of all participants are presented in Table 1, and details on the subjects’ medical history are provided in Supplementary Tables S3 and S4. Seronegative (*i.e*., never-infected) participants (group 1, *n* = 76; median [IQR] age, 44.5 [33–54] years; 37 women) and all recovered COVID-19 patients (groups 2 + 3, *n* = 69; 47 [36.5–52] years; 36 women) were similar in age (*p* = 0.532) and sex (*p* = 0.799). The median time interval between a positive COVID-19 antigen test and MRI was 157 [111.5–267.5] days. Subjects requiring inpatient treatment stayed in the hospital for a median of 9 [5.5–16.5] days, and 9/26 (34.6%) were treated in the intensive care unit for a median of 14 [5–53] days.

**Table 1:** Subject characteristics

Characteristic	Total	Control group	Patient groups	
Group classification		1	2	3
Number of individuals	145	76	43	26
Age, years, median [IQR]	46 [33.5–53]	44.5 [33–54]	42 [30–50]	51 [44.25–58.25]
Male, *n* (%)	72 (49.7)	39 (51.3)	18 (41.9)	15 (57.7)
Female, *n* (%)	73 (50.3)	37 (48.7)	25 (58.1)	11 (42.3)
Days after infection, median [IQR]	n/a	n/a	153 [106–302]	163 [112.75–219]
BMI, kg/m^2^, median [IQR]	24.88 [22.31–27.38]	24.08 [22.12–26.97]	25.06 [21.46–27.15]	26.74 [23.73–29.89]

BMI was calculated as weight in kilograms divided by height in meters squared. Age- and sex-matched controls form group 1; patients who recovered from COVID-19 at home form group 2; patients who were hospitalized due to COVID-19 form group 3

*BMI* Body mass index, *COVID-19* Coronavirus disease 2019, *IQR* Interquartile range, *n/a* Not applicable

When comparing subjects who had recovered from SARS-CoV-2 infection to controls, significant differences were observed in their ability to smell (*p* < 0.001), health-related quality of life (*p* = 0.001), sleepiness (*p* < 0.001), sleep quality (*p* < 0.001), and depression (*p* = 0.016). No significant differences were found for cognition and standardized neurological exam results. Ability to smell, sleepiness, and quality of sleep were significantly influenced only by a previous history of COVID-19 (odds ratios, 3.303 (95% confidence intervals, 1.727–6.474), *p* < 0.001; 0.357 (0.193–0.652), *p* = 0.001; 0.314 (0.167–0.584), *p* < 0.001), but not by age or gender. Reduction of health-related quality of life was significantly influenced by SARS-CoV-2 status (2.727 (1.453–5.186), *p* = 0.002) and to a lesser extent by age (0.961 (0.936–0.985), *p* = 0.002). Depression was also influenced by SARS-CoV-2 status (0.486 (0.266–0.883), *p* = 0.018) and additionally by gender (0.536 (0.293–0.971), *p* = 0.041).

**Fig. 1:**
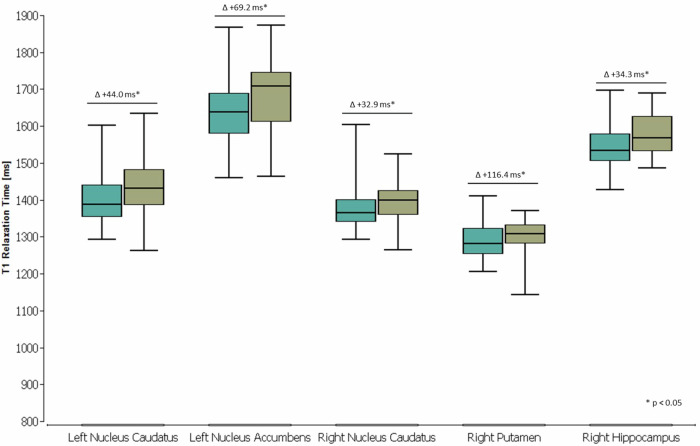
Box plots of qT1 values in five regions of interest, showing compelling trends of increased values in previously hospitalized COVID-19 patients *versus* controls, all of whom were unvaccinated and aged 40 years or older. Data were analyzed using Wilcoxon–Mann–Whitney *U* tests, stratified by age and controlled for false discoveries by the Benjamini-Hochberg method. Five deep gray matter regions (*x*-axis) showed significant differences (Δ) in qT1 values (*y*-axis; [ms]) between the subgroup of controls (*n* = 47; box plots further to the left) and recovered COVID-19 patients (*n* = 23; box plots further to the right). COVID-19, Coronavirus disease 2019; qT1, Quantitative T1 relaxation times

Age-stratified Wilcoxon–Mann–Whitney *U* tests (Table 2) revealed significant differences in qT1 values between the subgroups (*stratum* 1 and 2) of group 1 (47/76; 26 women) *versus* group 3 (23/69; 10 women), with both subgroups also similar in age (*p* = 0.675) and sex (*p* = 0.447). These differences (uncorrected *p*-values < 0.050) were present between ≥ 40-year-old controls and previously hospitalized individuals (*stratum* 2) in the following regions: right hippocampus (*p* = 0.020), right putamen (*p* = 0.041), right and left caudate nuclei (*p* = 0.027; *p* = 0.009), and left accumbens nucleus (*p* = 0.017) (Fig. 1). After controlling the false discovery rate, all of these results remained statistically significant. The left pallidum was the only region with a significant qT1 increase in previously hospitalized patients compared to controls within the < 40-year-old subgroup (*stratum* 1); however, the uncorrected *p*-value of 0.042 was deemed not statistically significant after *post hoc* analysis. No significant differences in qT1 were found between group 1 *versus* 2. The qT1 values of each deep GM structure did not correlate significantly with pre-existing conditions or results from dedicated tests. Furthermore, none of the qT1 values correlated significantly with the time interval between COVID-19 diagnosis and MR scan or with hospitalization time. ‘Loss of smell’ (44/69) as an initial symptom correlated negatively (ρ = -0.246, *p* = 0.042) with the qT1 values of the left caudate nucleus. Additionally, ‘fatigue’ (60/69) and ‘headache’ (53/69), also reported as initial symptoms, correlated negatively (ρ = -0.240, *p* = 0.047; ρ = -0.370, *p* = 0.002) with the qT1 values of the left accumbens nucleus. None of the other initial symptoms, nor any persistent symptoms during the brain MRI, showed significant relationships with any deep GM structure.

**Table 2:** Results of Wilcoxon–Mann–Whitney *U* tests for comparisons of qT1 values between controls and patient groups, stratified by an age cutoff of 40 years, and listed by all segmented deep gray matter structures

Group 1 *versus* Group 2					Group 1 *versus* Group 3				
Structure	*Stratum*	*n*	*m*	*p*-value	Structure	*Stratum*	*n*	*m*	*p*-value
Left nucleus accumbens	1	29	18	0.983	Left nucleus accumbens	1	29	3	0.872
	2	47	25	0.710		2	47	23	0.017*
Left hippocampus	1	29	18	0.878	Left hippocampus	1	29	3	0.075
	2	47	25	0.632		2	47	23	0.183
Left nucleus caudatus	1	29	18	0.569	Left nucleus caudatus	1	29	3	0.286
	2	47	25	0.632		2	47	23	0.009*
Left pallidum	1	29	18	0.431	Left pallidum	1	29	3	0.042#
	2	47	25	0.599		2	47	23	0.465
Left putamen	1	29	18	0.418	Left putamen	1	29	3	0.099
	2	47	25	0.692		2	47	23	0.090
Left thalamus	1	29	18	0.143	Left thalamus	1	29	3	0.419
	2	47	25	0.114		2	47	23	0.519
Right nucleus accumbens	1	29	18	0.336	Right nucleus accumbens	1	29	3	0.872
	2	47	25	0.818		2	47	23	0.123
Right hippocampus	1	29	18	0.294	Right hippocampus	1	29	3	0.722
	2	47	25	0.058		2	47	23	0.020*
Right nucleus caudatus	1	29	18	0.878	Right nucleus caudatus	1	29	3	0.821
	2	47	25	0.496		2	47	23	0.027*
Right pallidum	1	29	18	0.630	Right pallidum	1	29	3	0.316
	2	47	25	0.341		2	47	23	0.086
Right putamen	1	29	18	0.599	Right putamen	1	29	3	0.497
	2	47	25	0.883		2	47	23	0.041*
Right thalamus	1	29	18	0.406	Right thalamus	1	29	3	0.539
	2	47	25	0.535		2	47	23	0.488

Age- and sex-matched SARS-CoV-2-seronegative controls form group 1; patients who recovered from COVID-19 at home form group 2; patients who were hospitalized due to COVID-19 form group 3; *stratum* 1 indicates participants < 40 years old; *stratum* 2 indicates participants ≥ 40 years old; *n* indicates the number of participants in group 1; *m* indicates the number of participants in group 2 or 3

*COVID-19* Coronavirus disease 2019, *SARS-CoV-2* Severe acute respiratory syndrome coronavirus 2, *qT1* Quantitative T1 relaxation times

* *p*-values with statistical significance after *post hoc* correction

# *p*-values without statistical significance after *post hoc* correction

The regional volume estimates between the total patient group and their matched controls showed no significant differences. These comparisons (all *p*-values > 0.050) included the right and left hippocampi (medians, 3,872 *versus* 3,962 mm³ and 3,724 *versus* 3,819 mm³), right and left putamen (4,650 *versus* 4,759 mm³ and 4,676 *versus* 4,768 mm³), right and left caudate nuclei (3,538 *versus* 3,614 mm³ and 3,407 *versus* 3,478 mm³), right and left accumbens nuclei (461 *versus* 469 mm³ and 539 *versus* 561 mm³), right and left pallidum (1,687 *versus* 1,700 mm³ and 1,706 *versus* 1,742 mm³), and right and left thalamus (6,971 *versus* 7,073 mm³ and 7,130 *versus* 7,229 mm³). Similarly, no significant differences emerged when comparing the subgroup of subjects aged ≥ 40 years who had recovered from moderate-to-severe COVID-19 to the respective controls (all *p*-values > 0.050): right and left hippocampi (3,876 *versus* 3,958 mm³ and 3,716 *versus* 3,844 mm³), right and left putamen (4,465 *versus* 4,690 mm³ and 4,533 *versus* 4,700 mm³), right and left caudate nuclei (3,321 *versus* 3,555 mm³ and 3,182 *versus* 3,424 mm³), right and left accumbens nuclei (413 *versus* 444 mm³ and 523 *versus* 535 mm³), right and left pallidum (1,606 *versus* 1,718 mm³ and 1,654 *versus* 1,755 mm³), and right and left thalamus (6,746 *versus* 6,950 mm³ and 6,940 *versus* 7,074 mm³). TIV was also found to be nonsignificantly smaller in the total group of recovered COVID-19 patients compared to controls (means ± standard deviations, 1,055,503 ± 91,697 mm³ *versus* 1,062,817 ± 112,948 mm³), as well as in the aforementioned subgroup (1,053,356 ± 117,916 mm³ *versus* 1,054,586 ± 94,781 mm³). Given that TIV was also lower in the COVID-19 group, these apparent raw volume differences are expected and do not imply tissue loss without appropriate normalization. After applying TIV normalization to each regional volume, the previously nonsignificant differences remained largely consistent above the 0.05 significance level, with two exceptions: in the total patient group, only the left hippocampi showed a significant reduction in normalized volume (*p* = 0.006; previously *p* = 0.151), while in the relevant subgroup (*stratum* 2 of group 3), only the right caudate nucleus exhibited a significantly reduced normalized volume (*p* = 0.028; previously *p* = 0.150). Both findings remained statistically significant after controlling for multiple comparisons.

**Fig. 2:**
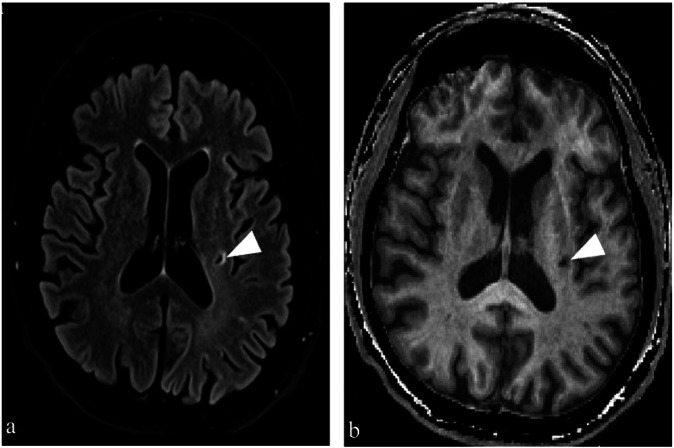
Representative case of a recovered COVID-19 patient with MRI-detectable macro- and microstructural brain changes. The 66-year-old male subject was hospitalized for over 3 months due to COVID-19. The MRI protocol included a conventional FLAIR sequence (**a**) and a synthetic anatomical MP-RAGE sequence (**b**), the latter of which was also used for software-based automatic segmentation of multiple deep gray matter structures. The visually interpreted images (**a**, **b**) revealed a lacuna in the left dorsal putamen (white arrowheads), which was excluded from the qT1 measurements (threshold exceeded). MRI qT1 maps showed increased qT1 values predominantly in the left caudate and accumbens nuclei (subject’s median qT1: 1,563 ms and 1,818 ms *versus* median qT1 of the never-infected controls aged ≥ 40: 1,398 ms and 1,644 ms), without any apparent lesions on conventional images. COVID-19, Coronavirus disease 2019; FLAIR, Fluid-attenuated inversion recovery; MP-RAGE, Magnetization-prepared rapid acquisition gradient-echo; MRI, Magnetic resonance imaging; qT1, Quantitative T1 relaxation times

Conventional neuroimaging revealed that the entire study population showed no evidence of chronic brain infarctions with a territorial pattern. In contrast, a higher percentage of lacunes was observed among all patients who had recovered from COVID-19 (5/69) compared to the never-infected population (0/76) (φ = 0.20; *p* = 0.023). This effect was even more pronounced in hospitalized (3/26) compared to nonhospitalized (2/43) patients and never-infected (0/76) subjects (φ = 0.24; *p* = 0.010). The lesions were located in the striatum (*n* = 3; areas = 3.2, 4.4, 6.1 mm²), and periventricular (*n* = 2; 17.2, 10.7 mm²) or deep (*n* = 1; 15.2 mm²) white matter. All three parenchymal defects located in the white matter were present in the subgroup of subjects older than 40 years who had required inpatient treatment. A representative case is shown in Fig. 2. The prevalence of WMH was as follows: 50.0% (38/76) for group 1, 58.1% (25/43) for group 2, and 38.4% (10/26) for group 3. There were no significant group differences in the ordinal grading scales of deep WMH indicative of SVD (*p* = 0.811). No significant associations were observed between groups (group 1 *versus* 2 *versus* 3) and the number of deep WMH.

## Discussion

The current study reports compelling trends of subtle tissue qT1 changes in specific deep brain regions in unvaccinated patients who recovered from COVID-19 during the pre-Omicron era. In contrast to home-recovered and younger subjects, patients aged ≥ 40 years who required hospitalization showed significantly increased qT1 values in the striatum (both-sided caudate nuclei, left-sided accumbens nucleus, and right-sided putamen) and the right-sided hippocampus compared to age- and sex-matched never-infected controls. These findings were independent of the time from original diagnosis, duration of inpatient treatment, and results from neuro-(psycho)logical tests, including cognitive assessment.

A body of scientific literature has reported that SARS-CoV-2 can trigger neuroinflammatory [32, 33], neurovascular [34, 35], and neurodegenerative [36, 37] processes during and even after acute infection. This study provides further insights into the microstructural changes of COVID-19 patients by imaging their brain microstructure with qT1 maps. Since changes in qT1 values are not pathognomonic for a specific underlying cause, they cannot be used to elucidate the origin of these changes. *Post-mortem* histopathological investigations have revealed edema, infiltration by inflammatory cells, activation of microglia, and astrogliosis in the basal ganglia, among other findings [38]. While qT1 increases may reflect any of these changes, they can also result from tissue loss due to atrophy that develops as a later consequence. After applying TIV normalization to control for inter-individual variability in head size, we identified two specific regions with significantly reduced normalized volumes: the left hippocampus in the total COVID-19 group, and the right caudate nucleus in the subgroup of patients aged ≥ 40 years after moderate-to-severe disease. Notably, only the right caudate nucleus showed this volume reduction in conjunction with elevated qT1 values. This combination may reflect microstructural damage consistent with a degenerative process, potentially not directly caused by the virus itself but rather indicative of downstream or secondary mechanisms. Neuropathologists have also described microbleeds in the basal ganglia and hippocampus [39]. However, superparamagnetic hemosiderin deposition would reduce qT1 values only if present in isolation without other contributing factors. Therefore, it should be noted that qT1 measurement alone, as performed in this study, is a suitable technique for detecting pathological changes but cannot distinguish between the various underlying processes.

Until now, studies of COVID-19 employing microstructural imaging with the use of automated preprocessing and image co-alignment for quantitative data have been notably uncommon. The impactful study by Douaud et al [40], which included diffusor tensor imaging in an elderly cohort of 401 patients *versus* 384 controls, did not reveal any changes in mean diffusivity, a surrogate marker for active neuronal degeneration, in the basal ganglia and hippocampi before *versus* after SARS-CoV-2 infection. In contrast, a study specifically focusing on subjects with post-COVID fatigue demonstrated changes in fractional anisotropy, another measure of diffusion and an indicator of nerve fiber integrity, within the thalami and basal ganglia [41]. Of note, metrics of diffusion tensor imaging and qT1 mapping rely on distinct physical processes and therefore measure different aspects of microstructural changes [42]. Moreover, the thalamus and basal ganglia regions showed decreased blood volumes in nonhospitalized patients [43]. Other types of functional imaging, such as voxel-based fluorine-18-fluorodeoxyglucose positron emission tomography, have indicated lower metabolic rates of glucose in the caudate nucleus after COVID-19 [44, 45].

Many reports have documented that COVID-19 can lead to macrostructural brain pathologies, such as ischemic and hemorrhagic stroke, cerebral venous and dural sinus thrombosis, meningitis, encephalitis, and diffuse edema [46–48], as well as WMH indicating brain inflammation. These findings are similar to those generally observed in viral respiratory infections, particularly in the thalamus [10] and hippocampus [49], as well as in the basal ganglia, such as the pallidum [50]. Similar to our findings, Shahjouei et al [46] reported lacunes after COVID-19, yet we cannot exclude the possibility of pre-existing silent strokes or in-hospital strokes resulting from therapeutic interventions. Additionally, our findings align with a meta-analysis investigating vascular-related WMH in COVID-19 patients, which revealed a lower prevalence in hospitalized patients compared to the crude rate [51]. Similarly, we observed lower proportions of deep WMH in COVID-19 survivors who required hospitalization compared to those who recovered at home and never-infected controls. In this study, we specifically addressed the deep WM, as deep WMH are more strongly associated with SVD, whereas periventricular WMH are more likely linked to nonischemic causes or chronic hemodynamic insufficiency resulting from carotid atherosclerosis [28]. The evolution of WMH is increasingly recognized as a multifactorial process, with age being a major risk factor. Depending on the cardiovascular risk profile, WMH are already common in midlife [52]. Up to the age of 40, the brain is still maturing, but its microstructure undergoes significant changes as part of the aging process [53]. Hagiwara et al reported an age-related increase in T1 relaxation times of the white matter and both cortical and deep GM after the age of around 60 [54]. It is important to note that vascular-related WMH can be smaller than 3 mm, raising the possibility that some lesions may have been overlooked on our two-dimensional FLAIR sequence during analysis due to their small size.

Studies investigating neurological and cognitive impairment in neurological disorders have focused on voxel-based morphometry, diffusion tensor imaging, and functional MRI, while MR relaxometry has fallen out of focus in recent years [40, 55–57]. In the 2010s, it became possible for the first time to detect diffuse damage in patients with multiple sclerosis using quantitative MR parameters, but the technique was largely abandoned due to inconsistent data. However, further technical developments in MR magnets and their gradient systems, as well as sequence developments in relaxometry, have enabled a renaissance of the method, especially for the detection of ‘invisible’ microstructural brain inflammation. In COVID-19 patients, changes in cortical thickness, mean diffusivity, blood oxygen level and fluorodeoxyglucose metabolism have been associated with cognitive decline [40, 44] and olfactory dysfunction [55]. Unlike those findings, our study did not show significant relationships between qT1 changes and focal neurological deficits, ability to smell, depression, sleepiness, sleep quality, health-related quality of life, or cognition. However, various COVID-19-associated symptoms correlated predominantly negatively with qT1 values in different brain regions. Whether these alterations were pre-existing or the result of COVID-19 remains unclear.

The limited sample size of our study is its main shortcoming. Additionally, we did not apply more conservative *p*-value corrections, such as the Bonferroni method, so this exploratory analysis may only indicate compelling trends. Our findings are based on a specific qT1 mapping approach, which may limit the direct generalizability of our results to other qT1 mapping techniques and, consequently, their broader applicability in clinical practice. TIV normalization is a recognized method for adjusting volumetric differences due to cranial size, enhancing the comparability of regional brain volumes across subjects. However, while TIV normalization accounts for overall head size differences, it may not fully eliminate variability in relative measures of regional brain volumes. This residual variability can arise from factors such as the methodological aspects of the normalization process. In addition to TIV normalization, we applied age- and gender-adapted grouping as a general normalization approach to further account for demographic influences. However, other potential confounding factors, such as lifestyle influences, which can affect brain volumes independently of TIV, remain a challenge. In general, our findings do not extend to individuals under the age of 18. The heterogeneity of our study population, including different SARS-CoV-2 variants and disease courses, may also have influenced the results. Finally, we cannot distinguish between pre-existing and disease-related brain alterations due to the absence of prior brain MRI with conventional and qT1 sequences. To our knowledge, however, this is the first study to employ advanced MRI techniques using qT1 mapping in COVID-19 patients and compare these values with macrostructural findings, neurological manifestations, and results from objective tests.

In conclusion, unvaccinated survivors of moderate-to-severe COVID-19 aged 40 years and older had increased qT1 values compared to unvaccinated controls in the striatum and hippocampus. These findings suggest microstructural changes, which were independent of the time from original diagnosis, duration of inpatient treatment, pre-existing conditions, or results from neuro-(psycho)logical and cognitive tests. Imaging the brain microstructure with qT1 provides valuable insights into brain involvement following SARS-CoV-2 infection.

## Supplementary information

**Additional file 1:** **Supplemental table S1.** Acquisition parameters for mapping qT1 values using the variable flip angle method, including additional B1 and B0 mapping. **Supplemental table S2.** Visual interpretation of deep WMH on FLAIR images. **Supplemental table S3**. Subject symptoms and pre-existing conditions. **Supplemental table S4.** WHO classification of the disease course in SARS-CoV-2-infected subjects, along with scaling criteria for groups 2 and 3 of the study population

## Data Availability

Data generated or analyzed during the study are available on request to the corresponding author.

## Abbreviations

CNS: Central nervous system
COVID-19: Coronavirus disease 2019
FLAIR: Fluid-attenuated inversion recovery
GM: Gray matter
IQR: Interquartile range
MRI: Magnetic resonance imaging
qT1: Quantitative T1 relaxation times
SARS-CoV-2: Severe acute respiratory syndrome coronavirus 2
SVD: Small vessel disease
TIV: Total intracranial volume
WMH: White matter hyperintensities

## Declarations

### Guarantor

The scientific guarantor of this publication is Prof Dr Elke Hattingen.
