# Supplementary material for: Impact on the microstructure of deep gray matter in unvaccinated patients after moderate-to-severe COVID-19: insights from MRI T1 mapping
Source: Eur Radiol Exp. 2025 Jul 5;9:63. doi: 10.1186/s41747-025-00598-7 (PMC12228859; doi:10.1186/s41747-025-00598-7)
Supplement: Supplementary file 1 — Additional file 1: Supplemental table S1. Acquisition parameters for mapping qT1 values using the variable flip angle method, including additional B1 and B0 mapping. Supplemental table S2. Visual interpretation of deep WMH on FLAIR images. Supplemental table S3. Subject symptoms and pre-existing conditions. Supplemental table S4. WHO classification of the disease course in SARS-CoV-2-infected subjects, along with scaling criteria for groups 2 and 3 of the study population [file 41747_2025_598_MOESM1_ESM.pdf]

Impact on the microstructure of deep gray matter in unvaccinated patients after moderate-to-severe COVID-19: insights from MRI T1 mapping

ELECTRONIC SUPPLEMENTARY MATERIAL

Magnetic resonance imaging protocol and image analysis

The variable flip angle method was used with a FLASH-EPI hybrid readout to improve the signal-to-noise ratio [1, 2]. This method is based on the acquisition of two gradient echo datasets with different excitation angles, resulting in different degrees of T1- and proton density (PD)-weighting, from which qT1 can be derived from the contrast differences. B1 was mapped as previously described in the literature [3]. To account for distortions of the static magnetic field (B0), a field mapping scan was added with the same coverage and spatial resolution as for B1 mapping. QT1 data were corrected for the influence of B0 distortions as described in the study by Gracien et al. [4]. The generation of synthetic MP-RAGE anatomical datasets from the qT1 maps was performed according to the study by Nöth et al. [5]. This requires additional PD maps. To improve data quality, pseudo-PD maps were derived for this purpose from the qT1 maps, according to the study by Volz et al. [6], and used in the calculation process [4]. The qT1 and PD maps computed from the above sequences were then used to directly derive synthetic MP-RAGE anatomical datasets. These are intrinsically corrected for inhomogeneities in the radiofrequency field used for signal transmission (B1) and nonuniformities in the receive coil profile, and they are anatomically aligned with the underlying qMRI datasets, allowing for high accuracy in qMRI analysis.

Statistical analysis

Since the median ages differed significantly between each group (1 *versus* 2 *versus* 3), the Wilcoxon-Mann-Whitney *U* tests were stratified by age, with a threshold set at 40 years. Correlations were analyzed using Pearson's correlation coefficients (r) for continuous data or Spearman's rank correlation coefficients (ρ) for noncontinuous data. Multivariable linear and binary logistic regression analyses were performed with backward stepwise selection (Wald) modeling to determine independent associations with qT1 values and MR macrostructural findings. Thresholds for the backward selection process included a *p*-value of 0.05 for entry and 0.10 for removal of a variable. Variance inflation factors were calculated beforehand, and variables with values greater than 10 were excluded to fit the statistical models by minimizing multicollinearity issues. Adjusted multivariate analyses were performed to determine whether age, gender, and SARS-CoV-2 status had any influence on ability to smell, sleepiness, quality of sleep, depression, cognition or health-related quality of life. The number of included subjects was estimated by an a priori sample size analysis under the assumption that groups would show a difference in qT1 values of 20 ms, resulting in a power of 79%.

References

1. Preibisch C, Deichmann R (2009) Influence of RF spoiling on the stability and accuracy of T1 mapping based on spoiled FLASH with varying flip angles. Magn Reson Med 61:125–135. https://doi.org/10.1002/mrm.21776

2. Preibisch C, Deichmann R (2009) T1 mapping using spoiled FLASH-EPI hybrid sequences and varying flip angles. Magn Reson Med 62:240–246. https://doi.org/10.1002/mrm.21969

3. Volz S, Nöth U, Rotarska-Jagiela A et al (2010) A fast B1-mapping method for the correction and normalization of magnetization transfer ratio maps at 3 T. Neuroimage 49:3015–3026. https://doi.org/10.1016/j.neuroimage.2009.11.054

4. Gracien R, van Wijnen A, Maiworm M et al (2019) Improved synthetic T1-weighted images for cerebral tissue segmentation in neurological diseases. Magn Reson Imaging 61:158–166. https://doi.org/10.1016/j.mri.2019.05.013

5. Nöth U, Hattingen E, Bähr O et al (2015) Improved visibility of brain tumors in synthetic MP-RAGE anatomies with pure T1 weighting. NMR Biomed 28:818–830. https://doi.org/10.1002/nbm.3324

6. Volz S, Nöth U, Jurcoane A et al (2012) Quantitative proton density mapping: correcting the receiver sensitivity bias via pseudo proton densities. Neuroimage 63:540–552. https://doi.org/10.1016/j.neuroimage.2012.06.076

Supplemental table S1. Acquisition parameters for mapping qT1 values using the variable flip angle method, including additional B1 and B0 mapping

Methods	FOV [mm³]	TR / TE_1/TE_2 [ms]	Flip angle α_1/α_2 [°]	BW [Hz/Pixel]	Voxel size [mm³]	Acquisition time [min:s]
VFA	256×224×160	16.4 / 6.7	4/24	222	1×1×1	9:48
B1 mapping	256×224×160	11 / 5	11	260	4×4×4	1:45
B0 mapping	256×224×160	560 / 4.89/7.35	60	200	4×4×4	1:03

VFA indicates the acquisition of two three-dimensional gradient-echo sequences that differ solely in their flip angles, enabling the quantification of T1 relaxation times through the contrast differences between the two acquisitions; B1 indicates the radiofrequency field; B0 indicates the static magnetic field. *BW* Bandwidth, *FOV* Field of view, *qT1* Quantitative T1 relaxation times, *TE* Echo time, *TR* Repetition time, *VFA* Variable flip angle.

Supplemental table S2. Visual interpretation of deep WMH on FLAIR images

Score	*Scaling criteria*
Grading deep WMH indicative of SVD using the Fazekas scale	
0	Absence
1	Punctate foci
2	Beginning confluence of foci
3	Large confluent areas
Grading the number of deep WMH indicative of SVD	
0	0
1	1 – 5
2	6 – 10
3	11 – 25
4	> 25

WMH White matter hyperintensities, *FLAIR* Fluid-attenuated inversion recovery, *SVD* Small vessel disease.

Supplemental table S3. Subject symptoms and pre-existing conditions

Category	Control group		Patient group				*p*-value	
**Group classification**	**1**		**2**		**3**			
Number of subjects (*n*)	76		43		26			
Time point	Before MRI*	During MRI**	During infection*	After infection**	During infection*	After infection**	during infection	after infection
**Symptoms, *n* (%)**	26 (34.2)	3 (4.0)	42 (97.7)	33 (76.7)	26 (100.0)	23 (88.5)	**< 0.001**	**< 0.001**
Loss/Impairment of taste, *n* (%)	0	0	26 (60.5)	5 (11.6)	12 (46.2)	3 (11.5)	**< 0.001**	**0.010**
Loss/Impairment of smell, *n* (%)	0	0	32 (74.4)	9 (20.9)	12 (46.2)	6 (23.1)	**< 0.001**	**< 0.001**
Fever, *n* (%)	0	0	20 (46.5)	0	22 (84.6)	1 (3.9)	**< 0.001**	0.101
Sore throat, *n* (%)	3 (4.0)	0	21 (48.8)	0	10 (38.5)	0	**< 0.001**	1.000
Limb pain, *n* (%)	2 (2.6)	1 (1.3)	26 (60.5)	3 (7.0)	14 (53.9)	0	**< 0.001**	0.126
Shortness of breath, *n* (%)	2 (2.6)	2 (2.6)	11 (25.6)	2 (4.7)	17 (65.4)	3 (11.5)	**< 0.001**	0.190
Pressure on the chest, *n* (%)	0	0	18 (41.9)	6 (14.0)	10 (38.5)	6 (23.1)	**< 0.001**	**< 0.001**
Dry cough, *n* (%)	2 (2.6)	0	20 (46.5)	1 (2.3)	22 (84.6)	1 (3.9)	**< 0.001**	0.288
Productive cough, *n* (%)	0	0	4 (9.3)	1 (2.3)	3 (11.5)	0	**0.016**	0.305
Fatigue, *n* (%)	8 (10.5)	0	36 (83.7)	24 (55.8)	24 (92.3)	16 (61.5)	**< 0.001**	**< 0.001**
Thrombosis, *n* (%)	0	0	0	0	0	0	1.000	1.000
Headache, *n* (%)	14 (18.4)	0	35 (81.4)	12 (27.9)	18 (69.2)	7 (26.9)	**< 0.001**	**< 0.001**
Conjunctivitis, *n* (%)	0	0	5 (11.6)	3 (7.0)	1 (3.9)	2 (7.7)	**0.010**	0.058
Diarrhea, *n* (%)	1 (1.3)	0	18 (41.9)	2 (4.7)	12 (46.2)	5 (19.2)	**< 0.001**	**< 0.001**
**Pre-existing conditions, *n* (%)**	45 (59.2)		35 (81.4)		23 (88.5)		0.004	
Neurological conditions, *n* (%)	0		4 (9.3)		3 (11.5)		0.016	
Asthma, *n* (%)	6 (7.9)		2 (4.7)		5 (19.2)		0.110	
Other chronic lung diseases, *n* (%)	1 (1.3)		1 (2.3)		0		0.725	
Heart conditions, *n* (%)	4 (5.3)		6 (14.0)		5 (19.2)		0.086	

Hypertension, *n* (%)		6 (7.9)		4 (9.3)		8 (30.8)		**0.008**
Diabetes mellitus II, *n* (%)		3 (4.0)		1 (2.3)		2 (7.7)		0.554
Cancer, *n* (%)		0		0		3 (11.5)		**< 0.001**
Pulmonary embolism, *n* (%)		0		0		1 (3.9)		0.101
Deep vein thrombosis, *n* (%)		0		1 (2.3)		0		0.305
Allergies, *n* (%)		24 (31.6)		20 (46.5)		11 (42.3)		0.242
Autoimmune diseases, *n* (%)		3 (4.0)		5 (11.6)		2 (7.7)		0.281
Rheumatoid arthritis, *n* (%)		2 (2.6)		0		3 (11.5)		**0.034**
Arthritis, *n* (%)		1 (1.3)		4 (9.3)		3 (11.5)		0.063

* indicates the time point within the last 6 months for the control group or during COVID-19 for the patient groups; ** indicates the time point at which the MRI was performed for both the control and patient groups. Neurological conditions were as follows: asymptomatic meningioma (*n* = 1), seizure during childhood (*n* = 2), migraine (*n* = 4). *COVID-19* Coronavirus disease 2019, *MRI* Magnetic resonance imaging.

Supplemental table S4. WHO classification of the disease course in SARS-CoV-2-infected subjects, along with scaling criteria for groups 2 and 3 of the study population

	Description	WHO Score	Group	Prevalence
Mild disease	Ambulatory, virus positive, no restriction of activities	1	2	9/69 (13.0%)
	Ambulatory, virus positive, restriction of activities	2		34/69 (49.3%)
Moderate disease	Hospitalisation, virus positive, no oxygen therapy	3	3	7/69 (10.1%)
	Hospitalisation, virus positive, oxygen by mask or nasal cannula	4		9/69 (13.0%)
Severe disease	Hospitalisation, virus positive, noninvasive ventilation or high flow oxygen	5		4/69 (5.8%)
	Hospitalisation, virus positive, intubation and mechanical ventilation	6		5/69 (7.3%)
	Hospitalisation, virus positive, ventilation plus additional organ support (pressors, RRT, ECMO)	7		1/69 (1.5%)

ECMO Extracorporeal membrane oxygenation, *MRI* Magnetic resonance imaging, *RRT* Renal replacement therapy, *SARS-CoV-2* severe acute respiratory syndrome coronavirus 2, *WHO* World Health Organization.
